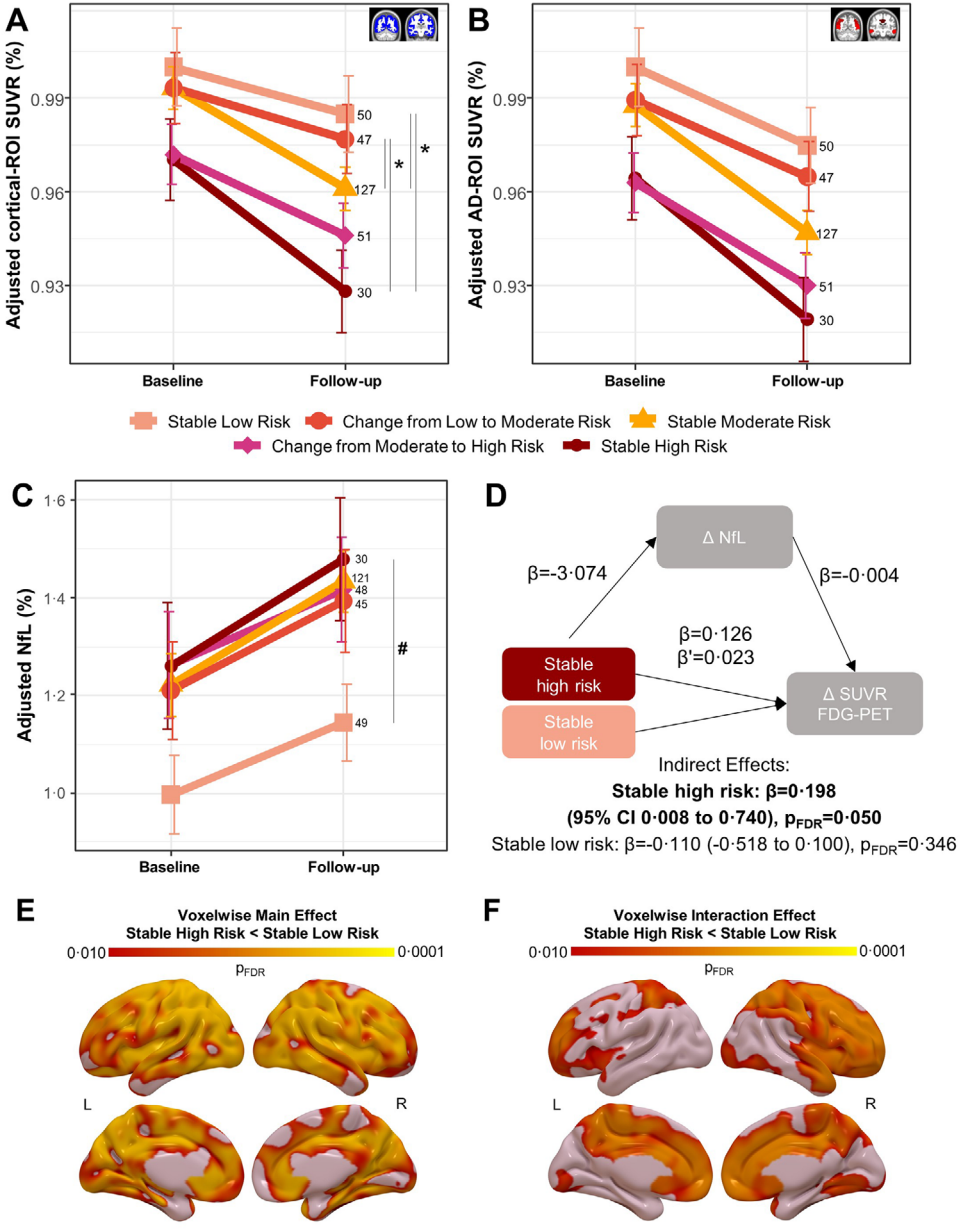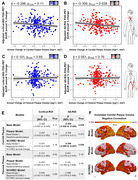# Longitudinal interplay between cerebral glucose metabolism, subclinical atherosclerosis and cardiovascular risk factors in midlife: the PESA study

**DOI:** 10.1002/alz.093794

**Published:** 2025-01-09

**Authors:** Catarina Tristão‐Pereira, Valentin Fuster, Ines Garcia‐Lunar, Michael Schöll, Marc Suarez‐Calvet, Maria Angeles Moro, Ana Garcia‐Alvarez, Javier Sanchez‐Gonzalez, Henrik Zetterberg, Kaj Blennow, Borja Ibañez, Juan Domingo Gispert, Marta Cortes‐Canteli

**Affiliations:** ^1^ Centro Nacional de Investigaciones Cardiovasculares (CNIC), Madrid Spain; ^2^ Icahn School of Medicine at Mount Sinai, New York, NY USA; ^3^ CNIC, Madrid, None Spain; ^4^ Wallenberg Centre for Molecular and Translational Medicine, University of Gothenburg, Gothenburg Sweden; ^5^ Barcelona?eta Brain Research Center (BBRC), Pasqual Maragall Foundation, Barcelona Spain; ^6^ Spanish National Center for Cardiovascular Research (CNIC), Madrid Spain; ^7^ CNIC, Madrid Spain; ^8^ Philips Healthcare Iberia, Madrid Spain; ^9^ Institute of Neuroscience and Physiology, Sahlgrenska Academy at the University of Gothenburg, Mölndal, Gothenburg Sweden; ^10^ Institute of Neuroscience and Physiology, Sahlgrenska Academy at the University of Gothenburg, Göteborg Sweden; ^11^ Spanish National Center for Cardiovascular Research, Madrid Spain; ^12^ Instituto de Investigación Sanitaria Fundación Jiménez Díaz (IIS‐FJD), Madrid Spain

## Abstract

**Background:**

Cardiovascular disease and dementia often co‐exist at advanced stages. Yet, midlife longitudinal studies examining the interplay between atherosclerosis and its risk factors on brain health are scarce. We aimed to determine the longitudinal associations between cerebral glucose metabolism, subclinical atherosclerosis and cardiovascular risk factors in middle‐aged asymptomatic individuals.

**Method:**

The Progression of Early Subclinical Atherosclerosis (PESA) is a longitudinal observational cohort study of 4184 asymptomatic individuals aged 40‐54. Participants with subclinical atherosclerosis underwent longitudinal cerebral [18F]fluorodeoxyglucose (FDG)‐PET (outcomes). Cardiovascular risk was quantified with SCORE2 and subclinical atherosclerosis with 3D vascular ultrasound (exposures). Multivariate regression and linear mixed effects models were used to assess outcome‐exposure associations through region‐of‐interest and voxelwise approaches. Additionally, blood‐based biomarkers of neuropathology were quantified and mediation analyses performed.

**Result:**

This longitudinal study included 370 participants (median age: 49·8 [IQR=46·1‐52·2] years; 84% men; follow‐up: 4·7 [4·2‐5·2] years). Persistent high‐risk of cardiovascular disease was associated with an accelerated decline of cortical FDG uptake compared to low‐risk (ß=‐0·008 [95% CI ‐0·013 to ‐0·002]; pFDR=0·040), with plasma neurofilament light chain, a marker of neurodegeneration, mediating this association by 20% (ß=0·198 [0·008 to 0·740]; pFDR=0·050) (Figure 1). Moreover, progression of subclinical carotid atherosclerosis was associated with an additional decline in FDG uptake in Alzheimer’s disease brain regions, not explained by cardiovascular risk (ß=‐0·269 [95% CI ‐0·509 to ‐0·027]; p=0·029) (Figure 2).

**Conclusion:**

Middle‐aged asymptomatic individuals with sustained cardiovascular risk and subclinical carotid atherosclerosis already present brain metabolic decline, suggesting that maintaining cardiovascular health during midlife may be key to reducing neurodegenerative disease burden later in life.